# Pharmacokinetic and Safety Profile of the Novel HIV Nonnucleoside Reverse Transcriptase Inhibitor MK-8507 in Adults without HIV

**DOI:** 10.1128/AAC.00935-21

**Published:** 2021-11-17

**Authors:** Wendy Ankrom, Deanne Jackson Rudd, Andrea Schaeffer, Deborah Panebianco, Evan J. Friedman, Charles Tomek, S. Aubrey Stoch, Marian Iwamoto

**Affiliations:** a Merck & Co., Inc., Kenilworth, New Jersey, USA; b Celerion, Inc., Lincoln, Nebraska, USA

**Keywords:** HIV-1, MK-8507, antiretroviral agents, clinical pharmacology, pharmacokinetics

## Abstract

MK-8507 is a novel HIV-1 nonnucleoside reverse transcriptase inhibitor in clinical development with potential for once-weekly oral administration for the treatment of HIV-1 infection. Two randomized, double-blind, placebo-controlled phase 1 studies in adults without HIV-1 evaluated the safety, tolerability, and pharmacokinetics of single and multiple doses of MK-8507; drug interaction with midazolam (a cytochrome P450 3A4 substrate) and food effect were also assessed. In study 1, 16 participants received oral ascending single doses of MK-8507 (2 to 400 mg) or placebo in an alternating fashion. In study 2, 24 participants received ascending single doses of MK-8507 (400 to 1,200 mg) or placebo and multiple doses (once weekly for 3 weeks) of MK-8507 (100 to 400 mg) or placebo. MK-8507 pharmacokinetics were approximately dose proportional at 2 to 1,200 mg. MK-8507 had a time to maximum concentration of 2 to 7 h and a mean terminal half-life of ∼58 to 84 h. MK-8507 doses of ≥100 mg achieved a plasma concentration at 168 h postdose (7 days) associated with antiviral efficacy. A high-fat meal had no clinically meaningful effect on MK-8507 pharmacokinetics, and MK-8507 400 mg once weekly had no clinically meaningful effect on midazolam pharmacokinetics. Single and multiple doses of MK-8507 were generally well tolerated. No trends with dose and no clinically meaningful changes were observed in vital signs, electrocardiograms, and laboratory safety tests. The pharmacokinetics and safety data are supportive of once-weekly oral administration and support further clinical investigation of MK-8507 for the treatment of HIV-1 infection.

## INTRODUCTION

Over the last 3 decades, substantial improvements have been made in oral HIV antiretroviral therapies (ART), which now offer people living with HIV (PLWH) the potential for a near-normal life expectancy (1, 2). To achieve this, individuals must maintain lifelong viral suppression, which requires daily administration of efficacious medication (3). Issues surrounding tolerability, complicated regimens, and treatment fatigue from daily dosing can lead to poor adherence and suboptimal viral suppression (3–6). Regimens can become complex when there is need to take multiple pills, requirements to take a medication fasted or with food, or potential for interactions with other medications, including those required to treat HIV-related comorbidities (3, 7, 8). New treatment options that are not only highly effective but also offer excellent tolerability, a high barrier to resistance, favorable drug interaction profiles, and the potential for less frequent dosing remain the focus of much clinical research (7, 9). While 1 pill once a day meets the needs of many PLWH, for others, daily administration poses challenges, including treatment fatigue and daily reminders and/or stigma associated with ART (10, 11). While treatment regimens that can be taken less often than daily are attractive to many PLWH, the long-acting injectable combination of cabotegravir-rilpivirine is currently the only treatment option available without daily dosing; however, administration requires injection by a health care professional (12), potentially posing other challenges.

MK-8507 is a novel, selective, potent HIV-1 nonnucleoside reverse transcriptase inhibitor (NNRTI) in clinical development with the potential for once-weekly (QW) oral administration, offering a unique treatment alternative for PLWH (13, 14; https://clinicaltrials.gov/ct2/show/NCT02174159). NNRTIs bind to a hydrophobic pocket in the p66 subunit of the p66/p51 heterodimer of the HIV-1 reverse transcriptase at a distance of 10 Å from the polymerase active site (15). MK-8507 is an allosteric inhibitor of HIV-1 reverse transcriptase that binds to the classic NNRTI hydrophobic binding pocket near the polymerase active site. MK-8507 has demonstrated *in vitro* activity against common NNRTI resistance-associated variants (16).

To evaluate the initial safety and pharmacokinetic (PK) profile of MK-8507, 2 phase 1 clinical trials were conducted in adults without HIV infection; a food effect assessment was also included. As *in vitro* data suggest the potential for MK-8507 to induce cytochrome P450 (CYP) 3A4, an enzyme often involved in drug metabolism (17, 18; https://www.fda.gov/drugs/drug-interactions-labeling/drug-development-and-drug-interactions-table-substrates-inhibitors-and-inducers#table2-1), an interaction assessment with the probe drug midazolam, a sensitive CYP3A4 substrate (https://www.fda.gov/drugs/drug-interactions-labeling/drug-development-and-drug-interactions-table-substrates-inhibitors-and-inducers#table2-1), was also conducted.

## RESULTS

A total of 16 participants were enrolled and completed study 1 (protocol MK-8507-001). A total of 24 participants were enrolled in study 2 (protocol MK-8507-002), and 23 participants completed the study per protocol; 1 participant withdrew for personal reasons. Participant demographics are summarized in Table 1.

**TABLE 1:** Participant demographics

Characteristic	Study 1 (*n* = 16)	Study 2 (*n* = 24)
Sex (no. [%])		
Male	16 (100.0)	21 (87.5)
Female	0 (0.0)	3 (12.5)
Age (mean [range] [yrs])	36 (24–53)	34 (21–54)
Race (no. [%])		
White	10 (62.5)	22 (91.7)
Black or African American	4 (25.0)	0 (0.0)
Black or African American, White	1 (6.3)	0 (0.0)
Asian	1 (6.3)	2 (8.3)
Ethnicity (no. [%])		
Not Hispanic or Latino/a	15 (93.8)	24 (100.0)
Hispanic or Latino/a	0 (0.0)	0 (0.0)
Unknown	1 (6.3)	0 (0.0)
Mean wt (range [kg])	83.5 (66.4–112.7)	83.7 (59.6–113.7)
Mean BMI^*a*^ (range [kg/m^2^])	26.47 (23.2–31.4)	26.3 (20.5–31.6)

a BMI, body mass index.

### Single-dose pharmacokinetics.

**FIG 1:**
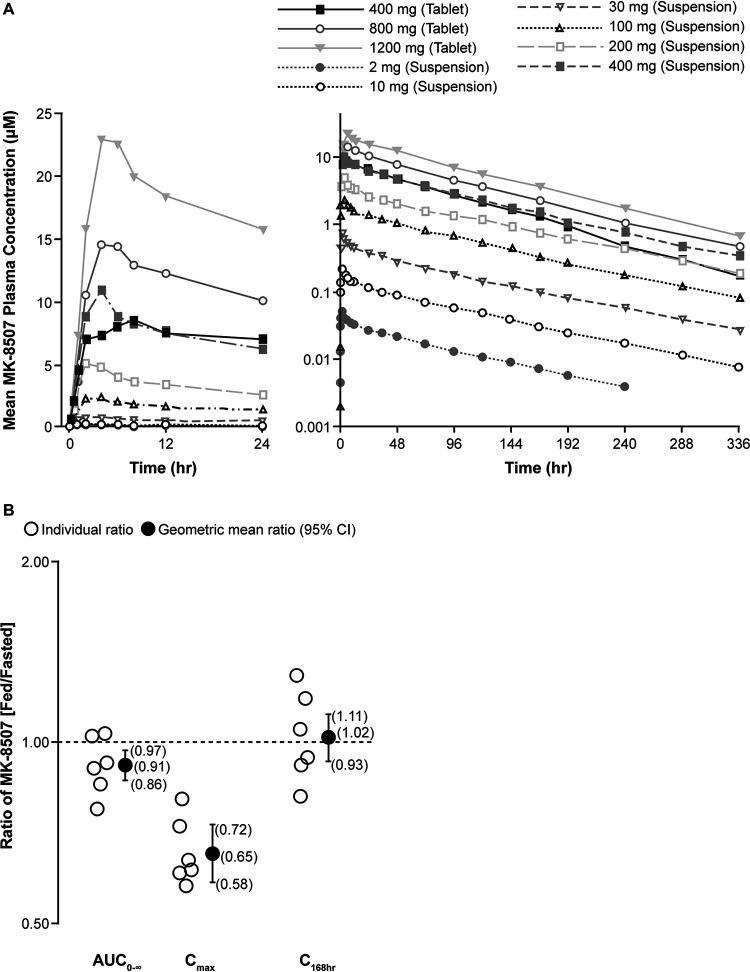
MK-8507 plasma PK following administration of single oral MK-8507 doses in adults without HIV. (A) Mean plasma concentration versus time profiles of MK-8507 following administration of single oral doses of MK-8507 suspension or tablet in the fasted state in adults without HIV (*n* = 6/dose level). (B) Individual MK-8507 plasma AUC_0–∞_, *C*_max_, and *C*_168hr_ ratios (fed/fasted) and GMRs with corresponding 90% CIs following the administration of a single oral dose of 100 mg MK-8507 suspension following a high-fat meal or following an overnight fast (*n* = 6) in adults without HIV. AUC_0–∞_, area under the concentration-time curve from 0 to infinity; *C*_168hr_, trough concentration; CI, confidence interval; *C*_max_, maximum concentration; GMR, geometric least-squares mean ratio.

The plasma MK-8507 concentration-time profiles and the plasma PK following single-dose administration of MK-8507 suspension and tablet formulations are summarized in Fig. 1 and Table 2. The mean terminal half-life (*t*_1/2_) ranged from 58 to 84 h. Comparing across doses, absorption following fasted administration of the suspension was faster (median time to maximum concentration [*T*_max_], 2 to 4 h) compared with the tablet (median *T*_max_, 4 to 7 h). A cross-study comparison of the 400-mg single-dose data was made; the geometric mean ratio (GMR; 90% confidence interval [CI]) of the tablet/suspension for area under the concentration-time curve from 0 to 168 h postdose (AUC_0–168hr_), maximum concentration (*C*_max_), and trough concentration (*C*_168hr_) were 0.95 (0.78, 1.17), 0.79 (0.60, 1.05), and 0.93 (0.66, 1.29), respectively, indicating similarity of the two formulations.

**TABLE 2:** Summary of MK-8507 plasma PK following administration of single oral MK-8507 doses of a suspension (2 to 400 mg) or tablet (400 to 1,200 mg) with fasting and 100-mg dose of suspension following a high-fat meal in adults without HIV^*b*^

Type of dose	Dose amt (mg)	No. of patients	AUC_0-∞_^*a*^ (geometric mean [95% CI] [μM·h])	AUC_0–168_^*a*^ (geometric mean [95% CI] [μM·h])	*C*_max_^*a*^ (geometric mean [95% CI] [μM])	C_168hr_^*a*^ (geometric mean [95% CI] [μM])	*T*_max_ (median [min, max] [h])	Apparent terminal *t*_½_ (geometric mean [%GVC] [h])
Suspension	2	6	3.82 (3.30–4.43)	2.94 (2.62–3.29)	0.05 (0.05–0.06)	0.01(0.01–0.01)	2.00 (2.00–2.00)	82.9 (8.54)
	10	6	15.2 (13.1–17.6)	11.9 (10.6–13.3)	0.22 (0.19–0.25)	0.03 (0.02–0.03)	2.00 (1.00–4.00)	82.0 (34.6)
	30	6	52.0 (44.9–60.2)	39.7 (35.4–44.6)	0.79 (0.68–0.90)	0.10 (0.08–0.13)	2.00 (2.00–4.00)	77.7 (9.97)
	100	6	175 (151–202)	139 (124–156)	2.58 (2.23–2.97)	0.30 (0.24–0.39)	2.04 (2.00–4.00)	75.9 (27.2)
	100 (fed)	6	159 (138–184)	126 (112–141)	1.67 (1.45–1.93)	0.31 (0.24–0.39)	10.00 (6.00–12.02)	72.6 (30.6)
	200	6	374 (323–433)	287 (256–322)	5.40 (4.68–6.23)	0.68 (0.53–0.87)	2.02 (2.00–4.03)	83.5 (14.3)
	400	6	770 (666–892)	619 (552–694)	11.5 (9.99–13.3)	1.30 (1.02–1.67)	4.00 (2.00–4.00)	69.1 (29.9)
Tablet	400	6	710 (592–850)	606 (501–732)	9.09 (7.13–11.6)	1.28 (0.99–1.64)	7.00 (2.00–12.00)	58.3 (14.9)
	800	6	1,230 (1,030–1,480)	989 (818–1,190)	15.4 (12.1–19.6)	2.10 (1.63–2.70)	4.00 (4.00–6.00)	70.9 (36.5)
	1,200	6	1,920 (1,600–2,300)	1,570 (1,300–1,890)	23.6 (18.5–30.1)	3.59 (2.79–4.61)	5.00 (4.00–8.00)	68.7 (12.8)

a Back-transformed LSM and CI from linear mixed-effects model performed on natural log-transformed values.

b AUC, area under the concentration-time curve; *C*_168hr_, trough concentration; CI, confidence interval; *C*_max_, maximum concentration; CV, coefficient of variation; GCV, geometric coefficient of variation; LSM, least-squares mean; max, maximum; min, minimum; *t*_1/2_, terminal half-life; *T*_max_, time to maximum concentration.

Plasma PK parameters (AUC_0–168hr_, *C*_max_, and *C*_168hr_) increased dose proportionally across the full range of 2 to 1,200 mg doses. The estimate of the slope of AUC_0–168_, *C*_max_, and *C*_168hr_ as a function of dose was close to 1, indicating dose proportionality (Table 3).

**TABLE 3:** Dose proportionality assessment of MK-8507 plasma pharmacokinetics following administration of single oral doses of 2 to 1,200 mg MK-8507 in participants without HIV^*b*^

Pharmacokinetic parameter	Estimate of slope	SE	95% CI for slope^*a*^
AUC_0–168hr_	1.0043	0.01042	(0.9869–1.0217)
*C*_max_	1.0227	0.02310	(0.9837–1.0618)
*C*_168hr_	0.9932	0.01638	(0.9655–1.0208)

a Confidence interval that includes 1.0 is consistent with dose proportionality.

b AUC_0–168hr_, area under the concentration-time curve from 0 to 168 h postdose; *C*_168hr_, trough concentration; CI, confidence interval; *C*_max_, maximum concentration.

### Food effect assessment.

A high-fat meal slightly delayed and reduced absorption of MK-8507 (Table 2 and Fig. 1B). A high-fat meal delayed MK-8507 *T*_max_ by ∼8 h and decreased *C*_max_ by 35% compared with fasted administration. However, the area under the concentration-time curve from 0 to infinity (AUC_0–∞_) and C_168hr_ were not meaningfully impacted; AUC_0–∞_ decreased by ∼9%, with no change in *C*_168hr_.

### Multiple-dose pharmacokinetics of MK-8507.

Plasma pharmacokinetics of MK-8507 following multiple-dose administration of MK-8507 tablet formulation are summarized in Table 4. Results were generally consistent with the single-dose data. Concentrations reached a median peak at ∼3 to 5 h postdose followed by a geometric mean apparent *t*_1/2_ ranging from 67 to 74 h. QW administration over 3 weeks of 100-mg, 200-mg, and 400-mg doses of MK-8507 resulted in minimal accumulation, with GMRs (day 36, day 22) of 1.18 to 1.23 for AUC_0–168hr_, 1.06 to 1.15 for *C*_max_, and 1.14 to 1.22 for *C*_168hr_.

**TABLE 4:** Summary of MK-8507 plasma pharmacokinetics following administration of multiple oral MK-8507 doses of a tablet (100 to 400 mg) administered once weekly for 3 weeks to adults without HIV^*c*^

Dose (mg)	Wk	No. of patients	AUC_0–168_^*a*^ (geometric mean [95% CI] [μM·h])	*C*_max_^*a*^ (geometric mean [95% CI] [μM])	*C*_168hr_^*a*^ (geometric mean [95% CI] [μM])	*T*_max_ (median [min, max] [h])	Apparent terminal *t*_½_^*b*^ (geometric mean [%GVC] [h])
100	1	6	157 (129–190)	2.94 (2.29–3.77)	0.32 (0.23–0.45)	3.00 (1.00–12.00)	
	2	6			0.39 (0.28–0.54)		
	3	6	185 (153–225)	3.29 (2.57–4.22)	0.37 (0.27–0.51)	3.03 (1.00–6.00)	66.7 (16.8)
GMR (accumulation ratio, wk 3/wk 1 [90% CI])		6	1.18 (1.12–1.25)	1.12 (0.96–1.30)	1.14 (0.87–1.50)		
200	1	5/6	285 (231–353)	4.87 (3.80–6.24)	0.59 (0.42–0.84)	5.00 (2.01–8.00)	
	2	5			0.70 (0.49–1.00)		
	3	5	352 (285–434)	5.61 (4.33–7.26)	0.72 (0.51–1.02)	4.01 (2.00–8.00)	74.2 (38.1)
GMR (accumulation ratio, wk 3/wk 1 [90% CI])		5/6	1.23 (1.16–1.31)	1.15 (0.98–1.36)	1.22 (0.90–1.65)		
400	1	6	563 (464–682)	10.8 (8.44–13.9)	1.23 (0.90–1.70)	4.00 (2.00–4.00)	
	2	6			1.11 (0.80–1.53)		
	3	6	694 (572–841)	11.5 (8.94–14.7)	1.49 (1.08–2.06)	4.00 (2.00–4.03)	73.1 (20.1)
GMR (accumulation ratio, wk 3/wk 1 [90% CI])		6	1.23 (1.16–1.31)	1.06 (0.91–1.23)	1.21 (0.92–1.59)		

a Back-transformed LSM and CI from linear mixed-effects model performed on natural log-transformed values.

b Geometric mean and percentage of geometric coefficient of variation reported for *t*_½_.

c AUC, area under the concentration-time curve; C_168hr_, trough concentration; CI, confidence interval; *C*_max_, maximum concentration; CV, coefficient of variation; GCV, geometric coefficient of variation; GMR, geometric mean ratio; LSM, least-squares mean; max, maximum; min, minimum; *t*_1/2_, terminal half-life; *T*_max_, time to maximum concentration.

### Midazolam drug-drug interaction assessment.

**FIG 2:**
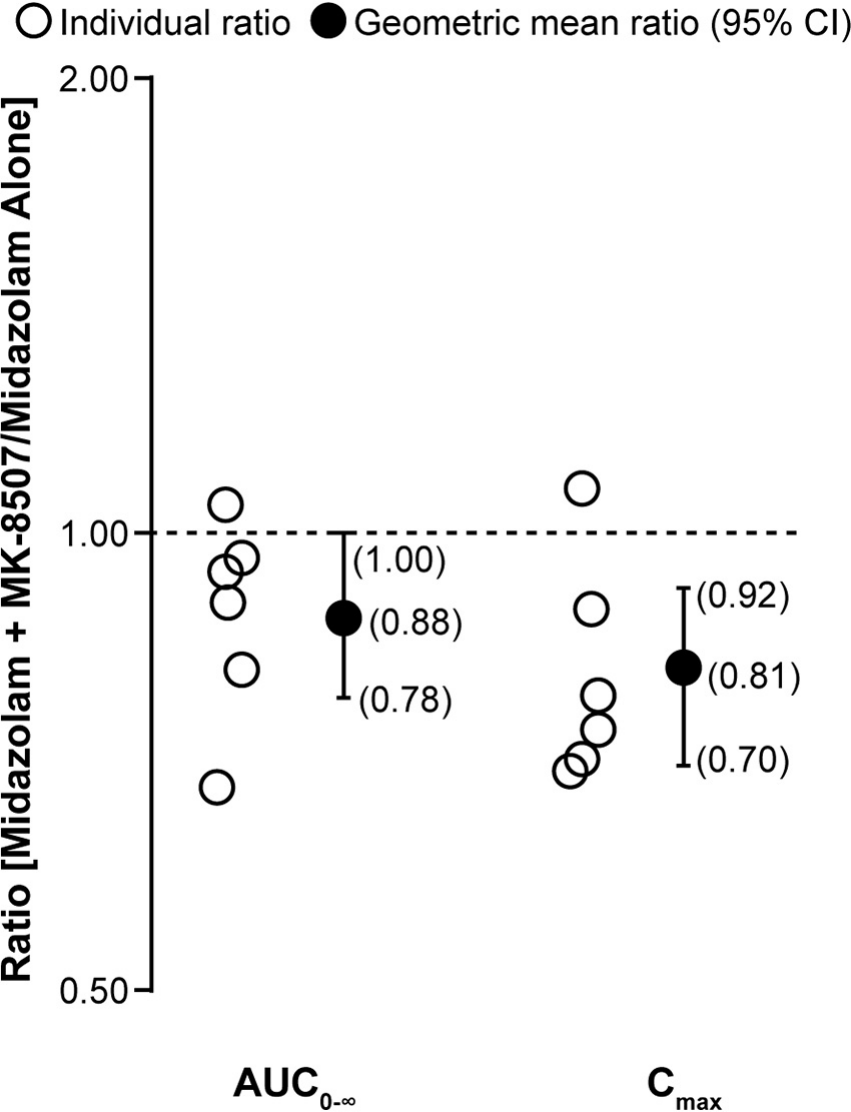
Individual midazolam AUC_0-∞_ and *C*_max_ ratios (midazolam plus MK-8507/midazolam alone) and GMRs with corresponding 90% CIs following administration of a single dose of 2 mg midazolam alone or with the third once-weekly oral 400-mg dose of MK-8507 (*n* = 6) in adults without HIV. AUC_0–∞_, area under the concentration-time curve from 0 to infinity; CI, confidence interval; *C*_max_, maximum concentration; GMR, geometric least-squares mean ratio.

The individual and geometric mean ratios of AUC_0–∞_ and *C*_max_ for midazolam with MK-8507 compared with midazolam alone are summarized in Fig. 2. Midazolam area under the concentration-time curve (AUC) and *C*_max_ decreased by 12% and 19%, respectively, when coadministered with MK-8507 dosed once weekly after 3 weeks.

### Safety and tolerability.

MK-8507 was generally well tolerated following a single dose as well as multiple QW doses. All adverse events (AEs) were nonserious and mild in intensity and resolved by the end of study, and no participants discontinued due to an adverse experience. No clinically meaningful relationships were observed for changes in clinical laboratory assessments, vital signs, or electrocardiograms following treatment with MK-8507.

In study 1, 12 participants (75.0%) reported a total of 38 nonserious AEs following treatment with a single oral dose of MK-8507, none of which were considered related to MK-8507. The most commonly reported AEs (≥2 participants) were headache (*n* = 4), cough (*n* = 3), myalgia (*n* = 2), and rhinorrhea (*n* = 2). In study 2, 6 participants (33.3%) reported a total of 10 nonserious AEs following single doses of MK-8507. One (decreased appetite) was considered related to MK-8507. Five participants (27.7%) reported a total of 13 nonserious AEs following multiple doses of MK-8507, none of which were considered related to MK-8507. No AE was reported by more than 1 participant. No trends were observed between the incidence of AEs and increasing dose levels in the single-dose or multiple-dose portions of the study.

## DISCUSSION

Maintaining viral suppression is central to positive health outcomes for PLWH (3, 19). A number of highly efficacious treatment options are available (19); however, there remains a disconnect between availability of effective treatments and effective disease control (5), which is partly driven by lack of adherence to ART regimens (4, 6). The novel NNRTI, MK-8507, a potentially highly potent, long-acting novel HIV-1 antiretroviral agent, is currently in clinical development as a QW oral treatment for HIV-1 infection. MK-8507 is being developed with the aim of providing a new treatment option for PLWH, with potential to address some of the limitations of current ART regimens (3, 7).

Two phase 1 clinical trials assessed the safety, tolerability, and PK profile of orally administered single and multiple doses of MK-8507 in adults without HIV-1 infection. MK-8507 was generally well tolerated, with no safety issues identified.

The plasma PK profile of MK-8507 is supportive of QW administration. Following oral administration, MK-8507 is readily absorbed, with a *T*_max_ of ∼2 to 7 h followed by a biphasic decline in plasma concentration. Terminal *t*_½_ is in the order of 70 h, with modest accumulation with multiple dosing. Across the doses studied, MK-8507 displayed approximately dose-proportional behavior. Concentration at trough is the PK parameter generally associated with antiviral efficacy of HIV treatment (20–23). Based on a meta-analysis of *C*_trough_ normalized by *in vitro* potency and viral load data from other NNRTIs (20), a PK target of *C*_trough_ ≥6 × 50% inhibitory concentration (IC_50_) is a threshold expected to achieve antiviral efficacy as part of combination therapy. PK results from these trials show that MK-8507 doses ≥100 mg meet or exceed this 6 × IC_50_ for MK-8507 threshold, 300 nM (148 ng/mL), at 7 days postdose (*C*_168hr_). These PK results support further development of MK-8507 as a QW oral agent for treatment of HIV administered as part of a complete treatment regimen together with islatravir. Evaluation of the antiviral efficacy of this regimen, including robustness under conditions of potential missed doses, is currently being evaluated in a phase 2 dose-ranging switch study of islatravir and MK-8507 QW in virologically suppressed adults with HIV (protocol MK-8591-013) (https://clinicaltrials.gov/ct2/show/NCT04564547).

A high-fat meal slowed absorption while having no meaningful effect on AUC_0–∞_ or *C*_168hr_. As efficacy concerns for HIV-1 NNRTIs are likely linked to decreases in *C*_trough_ rather than *C*_max_, the PK results indicate that MK-8507 may be dosed without regard to food. While the food effect assessment was conducted with the suspension formulation, the results can be applied to the tablet formulation given that the same active pharmaceutical ingredient form is present in both formulations. Additionally, MK-8507 pharmacokinetics in the fasted state were similar between the formulations, as demonstrated in the comparison at 400 mg. A lack of drug-food interaction is a highly desirable feature of an antiretroviral agent, providing maximal flexibility in dosing for PLWH (7, 8). While the food effect comparison was conducted with the oral suspension, cross-study comparison of the 400-mg dose indicated that while absorption was slower following tablet administration (median *T*_max_ was delayed, and *C*_max_ was reduced by 21% compared to suspension), AUC and *C*_trough_ were generally similar to the suspension. As AUC and *C*_trough_ are most closely linked with safety and efficacy, the food effect results with the suspension can be applied to the tablet formulation.

In preclinical *in vitro* studies, MK-8507 demonstrated the potential to induce expression of CYP3A4, which could lead to increased clearance of drugs metabolized by this pathway. Given that MK-8507 may be coadministered with drugs that are CYP3A4 substrates, an early evaluation of a potential effect of MK-8507 on this enzymatic pathway was warranted. Midazolam was selected as a probe substrate, as it is a substrate for CYP3A4-mediated metabolism recommended for use in index clinical drug-drug interaction studies (https://www.fda.gov/drugs/drug-interactions-labeling/drug-development-and-drug-interactions-table-substrates-inhibitors-and-inducers#table2-1). As induction can take from 7 to 14 days to reach steady state, midazolam was assessed with the third QW dose (i.e., after MK-8507 exposure had been present for 14 days) (24, 26). It was anticipated that any induction would have reached a maximum effect at the time of coadministration. Only a small decrease in midazolam plasma concentration was observed with coadministration (12% decrease in AUC and 18% decrease in *C*_max_) compared with midazolam administration alone. A weak inducer is characterized by a decrease in AUC of a sensitive index substrate by ≥20% to <50% (25). Based on these results, MK-8507 is not a meaningful inducer of CYP3A4 *in vivo*.

These trials have some limitations inherent to the nature of initial clinical investigation of a novel compound. Specifically, the study population was not entirely representative of PLWH. In particular, only males were enrolled in study 1 and only a small number of females in study 2. In addition, as MK-8507 possesses activity against HIV-1, the study was conducted in participants without HIV infection to prevent the risks associated with monotherapy resistance selection and to minimize confounders associated with HIV infection, comorbidities, and concomitant medications in PLWH. Nonetheless, data from this study are sufficient to support further development in PLWH, and efforts will be made to enroll a broader, more representative population in late-phase development. Another limitation is that some participants had *t*_1/2_ over 100 h. While the 336 h of PK sampling was long enough to capture approximately 5 half-lives of most participants, the estimates of *t*_1/2_ for those with significantly longer *t*_1/2_ values may have been less accurate.

In conclusion, MK-8507 PK and safety data support further clinical investigation of MK-8507. MK-8507 has the potential to be a highly potent HIV-1 NNRTI with favorable safety, tolerability, drug-drug interaction profiles, and PK properties that support QW administration. These features could fulfill the diverse needs of PLWH by providing a novel therapeutic option with less frequent administration, resulting in improved long-term treatment acceptance and adherence.

## MATERIALS AND METHODS

### Study design.

Two randomized, double-blind, placebo-controlled phase 1 clinical trials were conducted as outlined in Tables 5 and 6. Both trials were conducted at Celerion, Inc. (Lincoln, NE, USA) in conformance with good clinical practice standards and applicable local requirements regarding ethical committee review, informed consent, and other statutes or regulations regarding the protection of the rights and welfare of humans participating in biomedical research. The institutional review board for both studies was Chesapeake Research Review, Inc. (Columbia, MD, USA).

**TABLE 5:** Design of single rising-dose trial of MK-8507 (study 1)

Panel	No. of patients^*a*^	Treatment (MK-8507 dose or placebo) during:						
		Period 1		Period 2		Period 3		Period 4^*b*^
A	8	2 mg or placebo		30 mg or placebo		200 mg or placebo		
B	8		10 mg or placebo		100 mg or placebo^*b*^		400 mg or placebo	100 mg or placebo^*c*^ (with food)

a Randomized, double-blind, 3:1 active treatment:placebo.

b Final safety evaluation (21 days from last dose).

c Same participants received MK-8507 for crossover food effect assessment.

**TABLE 6:** Design of single and multiple once-weekly rising-dose trial of MK-8507 with midazolam interaction arm (study 2)

Panel	No. of patients^*a*^	Treatment at day −1	Single-dose treatment at:	Multiple-dose treatment at:	
			Day 1	Days 22 and 29	Day 36^*b*^
A	8		400 mg MK-8507 or placebo	100 mg MK-8507 or placebo	100 mg MK-8507 or placebo
B	8		800 mg MK-8507 or placebo	200 mg MK-8507 or placebo	200 mg MK-8507 or placebo
C	8	2 mg midazolam	1,200 mg MK-8507 or placebo	400 mg MK-8507 or placebo	400 mg MK-8507 or placebo and 2 mg midazolam

a Randomized, double-blind, 3:1 active treatment/placebo.

b Final safety evaluation (21 days from last dose).

Study 1 (protocol MK-8507-001) enrolled male participants without HIV-1 infection, ≥19 and ≤55 years of age, with a body mass index (BMI) of ≤32 kg/m^2^. Study 2 enrolled male and female participants without HIV-1 infection, ≥19 and ≤55 years of age, with a BMI of ≥18 kg/m^2^ and ≤32 kg/m^2^. In both studies, participants were required to be in good health without a history of significant abnormalities or diseases and were required to refrain from the use of any medication, including prescription and nonprescription medication and herbal remedies.

Study 1 was a 4-period, alternating panel (A and B), rising single-dose trial. Participants in each panel were randomized to receive single rising doses of MK-8507 (*n* = 6 per panel) or placebo (*n* = 2 per panel) as an oral suspension. All study drugs were administered after an overnight fast, except in period 4. The same participants who received 100 mg MK-8507 in period 2 received 100 mg MK-8507 in period 4 following a high-fat breakfast (55.6 g fat and approximately 850 kCal]). There was a minimum 20-day washout interval between doses within each panel and a 10-day interval between each dosing period.

Study 2 (protocol MK-8507-002) was a randomized, serial, 3-panel (A to C), single- and multiple-QW rising-dose trial. Single doses of 400, 800, and 1,200 mg MK-8507 or placebo tablet formulation were administered on day 1 to panels A, B, and C, respectively, to continue dose escalation beyond the dose range in study 1. Following a washout period of 3 weeks, the multiple-dose portion of the trial began. Participants in panels A, B, and C received QW doses of 100, 200, or 400 mg MK-8507 or placebo for 3 weeks (i.e., on days 22, 29, and 36), respectively. Participants were randomized such that 6 received MK-8507 and 2 received placebo in each panel; the same participants who received MK-8507 on day 1 also received MK-8507 on days 22, 29, and 36. The PK profile of midazolam following a single oral dose alone or after multiple oral dose administration of MK-8507 was also evaluated. In panel C, participants received 2 mg midazolam administered as a 2 mg/mL oral HCl syrup on day −1 and then coadministered with the third QW dose of MK-8507 on day 36. A 2-mg dose of midazolam was considered adequate to assess drug-drug interaction while being substantially lower than doses typically required for effective sedation (https://www.rxlist.com/midazolam-hydrochloride-syrup-drug.htm). At least 10 days separated dosing of 1 panel and initiation of the subsequent panel. In both studies, dose escalation decisions were based on a review of safety data and available PK data.

### Pharmacokinetics.

Blood samples for the determination of MK-8507 plasma concentrations were collected up to 336 h postdose (study 1 and study 2, day 1 and day 36) and up to 168 h postdose (study 2, day 22). Blood samples for determination of midazolam plasma concentrations were collected for up to 24 h postdose on day −1 and 48 h postdose on day 36. Plasma PK parameters evaluated for MK-8507 included AUC_0–∞_ (single dose only), AUC_0–168hr_, T_max_, *C*_max_, *C*_168hr_, and apparent terminal *t*_½_. Plasma PK parameters estimated for midazolam were AUC_0–∞_, *T*_max_, *C*_max_, and *t*_½_.

MK-8507 in plasma was extracted by protein precipitation and analyzed by reversed-phase chromatographic separation coupled with tandem mass spectrometric detection by Merck & Co., Inc. (West Point, PA, USA). The liquid chromatography-tandem mass spectrometric (LC-MS/MS) system consisted of a Waters Acquity ultraperformance liquid chromatography system (Waters Corporation, Milford, MA, USA) employing a turbo ionspray interface in the negative ion mode. The lower limit of quantitation was 1.0 ng/mL (2.03 nM) with a linear calibration range from 1.0 to 1000 ng/mL. The mean intrarun accuracy was 94.8 to 113% (precision, ≤6.2% coefficient of variation [CV]), and the mean interrun accuracy was 94.4 to 103.0% (precision, ≤8.6% CV).

Plasma samples collected for midazolam assay were analyzed by Syneos Health (Québec, Canada). The analytical method used liquid-liquid extraction for analyte isolation followed by LC-MS/MS detection. The lower limit of quantitation was 20.00 pg/mL, and the analytical range was 20.00 to 20,000.00 pg/mL.

### Safety and tolerability.

The safety and tolerability of MK-8507 were assessed by clinical evaluation, including vital signs, 12-lead electrocardiograms, laboratory safety tests, and clinical monitoring of AEs.

### Statistical analyses.

PK parameter values were calculated by noncompartmental analyses using the software Phoenix WinNonlin Professional (version 6.3) (Certara, Princeton, NJ, USA). *C*_max_, *C*_168hr_, and *T*_max_ were generated from the observed plasma concentration-time data. AUC_0–∞_ and AUC_0–168hr_ were calculated using the linear-trapezoidal method for ascending concentrations and the log-trapezoidal method for descending concentrations (linear up/log down). For each participant, the apparent terminal rate constant (λz) was calculated by regression of the terminal log-linear portion of the plasma concentration-time profile, and the apparent terminal *t*_½_ was calculated as the quotient of the natural log of 2 (ln[2]) and λz.

Statistical analyses were performed using SAS software version 9.3 (SAS Institute Inc., Cary, NC, USA). To evaluate the pharmacokinetics of single-dose MK-8507, values for MK-8507 PK parameters were natural log (ln) transformed and analyzed based on either a linear mixed-effects model containing a fixed effect for treatment and a random effect for participant (study 1) or a linear model containing a fixed effect for treatment (study 2). The least-squares geometric mean (GM) and corresponding 95% CIs obtained from the models were exponentiated to provide estimates for the population GMs and CIs on the original scales. To assess the effect of food, 90% CIs were calculated for the GMR (fed/fasted) of MK-8507 AUC_0-∞_, *C*_max_, and *C*_168hr_. To assess the effect of formulation, AUC_0–168hr_, *C*_max_, and *C*_168hr_ for the 400 mg suspension dose (study 1) and the 400-mg day 1 tablet dose (study 2) were ln transformed and analyzed using a linear model containing a fixed effect for formulation. The point estimates and the corresponding 95% CIs were obtained from the model for GM of each formulation; the GMR and 90% CIs comparing tablet to suspension were estimated using the model.

To assess dose proportionality, AUC_0–168hr_, *C*_max_, and *C*_168hr_ for single doses from 2 to 1,200 mg were ln transformed and analyzed using a linear mixed-effect model with ln(dose) as a covariate, group (i.e., study 1 panel A, study 1 panel B, and study 2) as a fixed effect, and participant as a random effect. The AUC_0–168hr_ and C_168hr_ group was not statistically significant at the 0.05 level and was therefore omitted from the final model. An overall slope was estimated across all groups. To evaluate the pharmacokinetics of multiple-dose MK-8507, PK parameters were ln transformed and analyzed using a linear mixed-effects model containing fixed effects for treatment, day and treatment-by-day interaction, and a random effect for participants. A 95% CI was constructed for the GM of MK-8507 AUC_0–168hr_, and *C*_max_ at each multiple-dose level following the day 22 (week 1) and day 36 (week 3) doses and MK-8507 *C*_168hr_ at each multiple-dose level following the day 22 (week 1), day 29 (week 2), and day 36 (week 3) doses. Accumulation of MK-8507 was assessed through the construction of a 90% CI for the GMR (week 1/week 3) of the AUC_0–168hr_, *C*_max_, and *C*_168hr_.

To assess the pharmacokinetics of midazolam, AUC_0–∞_ and *C*_max_ for midazolam were ln transformed and analyzed using a linear mixed-effects model containing a fixed effect for day (day −1 and day 36), and a random effect for participant. The PK profile of midazolam alone versus coadministration with 400 mg MK-8507 on day 36 was assessed through the construction of a 90% CI for the GMR (day 36/Day −1) of AUC_0–∞_ and *C*_max_.
